# A meta-analysis: elucidating diagnostic thresholds of peak systolic flow velocities in thyroid arteries for the discrimination of Graves’ disease and destructive thyrotoxicosis

**DOI:** 10.3389/fendo.2024.1393126

**Published:** 2024-06-07

**Authors:** Sinong Li, Zheng Ding, Xiang Li, Miao Fu, Li Sang, Mingxia Yang, Rubo Tang, Tianxiang Gu, Liang Sang

**Affiliations:** ^1^ Department of Ultrasound, The First Hospital of China Medical University, Shenyang, Liaoning, China; ^2^ Department of Cardiac Surgery, The First Hospital of China Medical University, Shenyang, Liaoning, China; ^3^ Department of Acupuncture and Massage, Shouguang Hospital of Traditional Chinese Medicine, Shouguang, China; ^4^ Department of Ultrasound, Shouguang People’s Hospital, Shouguang, China; ^5^ Department of Cardiology, Shouguang People’s Hospital, Shouguang, China

**Keywords:** graves’ disease, destructive thyrotoxicosis, superior thyroid artery, inferior thyroid artery, peak systolic velocities

## Abstract

**Objective:**

This meta-analysis examines peak systolic velocities (PSVs) in thyroid arteries as potential biomarkers for thyroid disorders, which includes treated and untreated Graves’ disease(GD) and destructive thyrotoxicosis(DT).

**Methods:**

A search across databases including PubMed, Google Scholar, Embase, and Web of Science identified studies assessing peak systolic flow velocity in the inferior thyroid artery (ITA-PSV) and superior thyroid artery (STA-PSV) diagnostic efficacy in GD and DT.And the search was restricted to publications in the English language.The analysis compared STA-PSV and ITA-PSV across patient groups, evaluating intra-group variances and synthesizing sensitivity and specificity data.

**Results:**

The analysis covered 18 studies with 1276 GD, 564 DT patients, and 544 controls. The difference of STA-PSV between GD group, DT group and normal group and the difference of ITA-PSV were analyzed in subgroups, and there was no statistical significance between subgroups when comparing any two groups. Normal subjects displayed intra-group ITA-PSV and STA-PSV differences with established cut-off values of 20.33 cm/s (95% CI, 17.48-23.18) for ITA-PSV and 25.61 cm/s (95% CI, 20.37-30.85) for STA-PSV. However, no significant intra-group differences were observed in the STA-PSV and ITA-PSV cut-off values among groups with GD or DT. The combined cut-off values for these patient groups and normal subjects were 68.63 cm/s (95% CI, 59.12-78.13), 32.08 cm/s (95% CI, 25.90-38.27), and 23.18 cm/s (95% CI, 20.09-26.28), respectively. The diagnostic odds ratio(DOR) for these values was 35.86 (95% CI, 18.21-70.60), and the area under the summary receiver operating characteristic (SROC) curve was 0.91, with a sensitivity estimate of 0.842 (95% CI, 0.772-0.866).

**Conclusion:**

PSVs in thyroid arteries are useful diagnostic tools in distinguishing DT from GD. A PSV above 68.63 cm/s significantly improves GD diagnosis with up to 91% efficacy. No notable differences were found between superior and inferior thyroid arteries in these conditions.

## 1 Introduction

Graves‘ disease, an autoimmune thyroid disorder predominantly mediated by T cells, presents a significant clinical challenge due to its prevalence across various age groups, particularly in women of childbearing age (1, 2). This condition, characterized by an inflammatory response in the thyroid gland, can be triggered by factors such as autoimmunity, infection, or certain medications. Among the autoimmune causes, destructive thyrotoxicosis encompasses a spectrum of disorders including Hashimoto thyroiditis (chronic lymphocytic thyroiditis), atrophic thyroiditis, painless thyroiditis, and postpartum thyroiditis. GD and Hashimoto’s thyroiditis emerge as the most common autoimmune thyroid disorder (3), with GD being a leading cause of hyperthyroidism (1). However, the clinical, biochemical, and ultrasonographic similarities between GD and DT often complicate differential diagnosis and treatment planning, thereby impacting patient prognosis and disease progression (4).

Currently, the preliminary diagnosis of hyperthyroidism is based on elevated serum levels of triiodothyronine (T3) and thyroxine (T4), coupled with reduced serum TSH levels. Advanced diagnostic confirmation involves assessing TSH receptor antibodies (TRAb), thyroid peroxidase antibodies (TPOAb), technetium 99m scanning, radioactive iodine uptake (RAIU), and thyroid ultrasound. However, these techniques have limitations in practical application. For instance, RAIU, while being a gold standard for etiological determination of thyrotoxicosis, is cumbersome, time-intensive, and susceptible to interference from medications and dietary factors (5). Technetium-99m scanning, though effective, is costly and not recommended for pregnant or breastfeeding women (6). Biochemical indices often present challenges in terms of interaction and stability.

Thyroid ultrasonography, particularly when combined with grayscale and color Doppler, offers real-time visualization of thyroid blood flow and structural changes (7), but results can be influenced by the operator’s experience and technique. Despite these challenges, thyroid ultrasonography is considered one of the most effective imaging modalities for thyroid assessment (4, 8, 9). The quantification of ITA-PSV and STA-PSV is a specific and sensitive method for differentiating GD from thyroiditis (5, 10, 11). Numerous studies have underscored the significance of peak systolic flow velocity in thyroid arteries for the diagnosis, differential diagnosis, and treatment of GD. For example, Saleh et al. demonstrated the efficacy of Doppler ultrasound in predicting the prognosis of GD and aiding in treatment strategy formulation (12). ITA-PSV has been proposed as a potential predictor of GD recurrence (13, 14), and the flow characteristics in the superior and inferior thyroid arteries have been identified as valuable markers for distinguishing GD from thyroiditis (4, 11, 15–18). Recent advancements have further highlighted the diagnostic value of peak systolic flow velocity in thyroid arteries, as evidenced by Peng et al.’s investigation into the sensitivity and specificity of STA-PSV cut-off values (19).

Despite the clinical importance of these parameters in predicting, treating, and differentiating GD, the precise diagnostic threshold for peak systolic flow velocity in thyroid arteries remains ambiguous, and the optimal measurement site is yet to be determined. This meta-analysis aims to evaluate the selectivity of superior versus inferior thyroid artery measurements in clinical practice, the establishment of diagnostic thresholds, and their potential in differentiating GD from DT.

## 2 Method

Following the PRISMA standards for systematic reviews and meta-analyses, this investigation was carried out (20).

### 2.1 Search strategy

Our comprehensive literature search spanned two decades, from 2003 to 2023, encompassing a range of databases including PubMed, Google Scholar, Embase, and Web of Science. The search was meticulously conducted using a detailed formula: (“peak systolic velocity of superior thyroid artery” OR “STA-PSV” OR “color flow doppler sonography” OR “doppler sonography” OR “ultrasonography” OR “echography” OR “ultrasound” OR “Blood Flow Velocities” OR “Flow Velocities, Blood” OR “Flow Velocity, Blood” OR “Velocities, Blood Flow” OR “Velocity, Blood Flow” OR “Superior thyroid artery” OR “inferior thyroid artery”) AND (“Graves’ disease” OR “GD” OR “Disease, Graves” OR “Disease, Graves’” OR “Hyperthyroidism, Autoimmune” OR “postpartum thyroiditis” OR “subacute thyroiditis” OR “painless thyroiditis”). The search was restricted to publications in the English language.

### 2.2 Inclusion and exclusion criteria

The inclusion criteria were: (1) diagnosis of patients confirmed through serologic testing, including FT3, FT4, TSH, and TRAb; (2) studies with a minimum of 20 cases of GD; (3) availability of STA-PSV or ITA-PSV measurements; and (4) clear categorization of study participants into groups with GD, DT, and normal controls. Exclusion criteria encompassed: (1) non-original data; (2) studies with missing or incomplete critical data; (3) duplicate publications in consecutive years in the same journal; and (4) studies with evident bias or irrational methodologies.

### 2.3 Article filtering and data extraction

The initial phase involved screening titles and abstracts for relevance to Graves’ disease and STA-PSV or ITA-PSV. Full texts were then obtained and further assessed against the inclusion and exclusion criteria. In cases of uncertainty, team discussions were conducted to reach a consensus on inclusion. Data extracted from each article included the first author, publication year, linear transducer frequency, number of subjects in each group, and raw data on peak systolic flow velocities in the superior or inferior thyroid arteries.

### 2.4 Data processing

Participants were categorized into three groups: those with GD, those with DT including Hashimoto’s thyroiditis, postpartum thyroiditis, and painless thyroiditis, and Normal. Statistical heterogeneity between studies was evaluated using the I² statistic (I² > 25% indicating low heterogeneity, I² > 50% moderate heterogeneity, and I² > 75% significant heterogeneity). A fixed-effects model was applied in cases of acceptable heterogeneity (P > 0.05, I² < 50%); otherwise, a random-effects model was utilized. For specific studies using interquartile range and median instead of mean and standard deviation, the Luo and Wang data conversion tool was employed (21–24). All analyses were conducted using R Studio software (version 4.1.2), with the ‘meta’ and ‘metafor’ packages (25, 26).

## 3 Result

### 3.1 Search results and study characteristics

**Figure 1:**
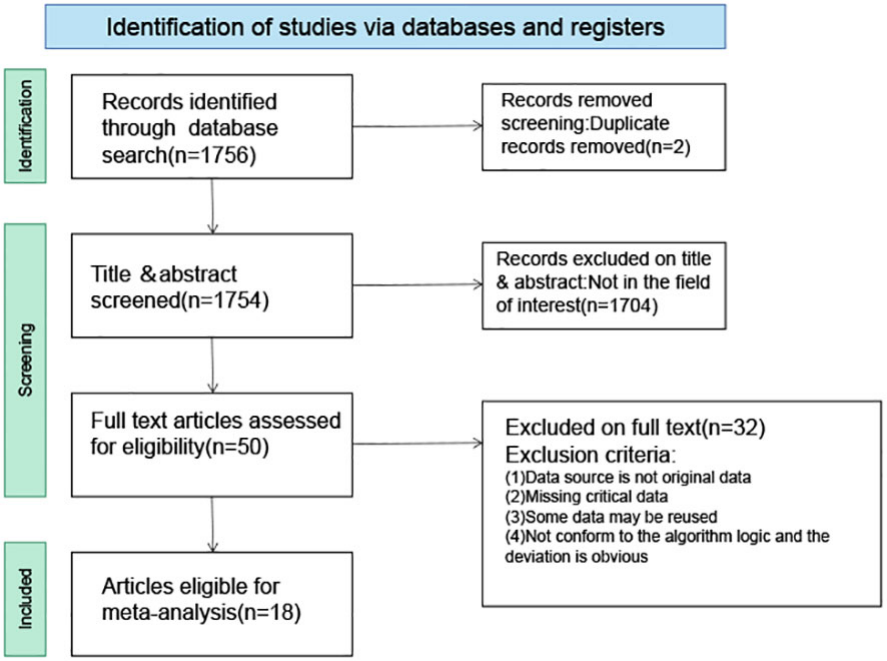
Literature screening strategy map.

**Figure 2:**
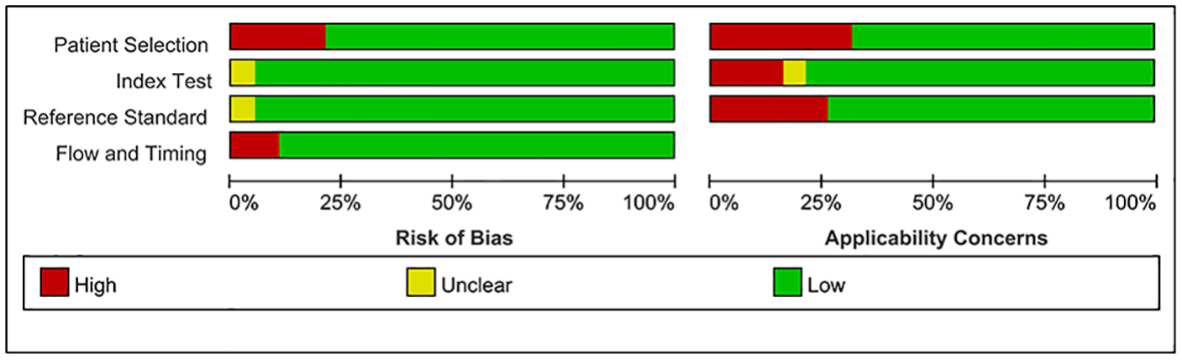
Document quality assessment map.

Employing a meticulously crafted search algorithm, we successfully retrieved 1,756 documents from various databases spanning from 2003 onwards. After a rigorous screening process, which involved the removal of 2 duplicates and the exclusion of 1,704 documents based on title and abstract evaluations, 50 documents were selected for full-text review. Ultimately, 18 studies (4–6, 10, 11, 13–16, 27–35) met our stringent criteria for inclusion in this meta-analysis. The process of literature screening and quality assessment is visually depicted in **Figures 1** and **2**, respectively. Data were extracted from 20 trials involving 1276 patients with GD, 564 patients with DT, and 544 normal participants. These subjects were diagnosed using serum tests such as FT3, FT4, TSH, and TRAb, as reported in the 18 selected studies. The baseline characteristics of the study are detailed in **Table 1**, while the STA/ITA-PSV data, obtained via ultrasonography, are presented in **Supplementary Tables 1** and **2**, and **Supplementary Figure 1**.

**Table 1:** Characteristics of participants included in the study,(1) retrospective study, (2) prospective study.

Author/Year	Sample size GD/DT/Normal	Gender(Male/Female)			Age		
		GD	DT	Normal	GD	DT	Normal
Ueda, 2005	79/NA/17	14/65	NA	4/13	41.3 ± 14.4	NA	39.5 ± 16.0
Nagasaki, 2007	47/NA/22	6/41	NA	3/19	35.2 ± 3.7	NA	40.8 ± 3.9
Nagasaki, 2010	42/NA/42	NA/42	NA	NA/32	34.7 ± 0.92	NA	35.7 ± 1.2
Banaka, 2013	29/70/48	7/22	7/63	11/37	41.5 ± 14.7	44.1 ± 12.4	43.3 ± 17.7
Zuhur, 2014	150/79/71	39/111	21/58	28/43	38.4 ± 12.8	39.2 ± 14.1	35.8 ± 10.5
Malik, 2019	46/19/NA	17/29	4/15	NA	35.26 ± 13.06	31.21 ± 8.87	NA
Santos, 2020	97/NA/NA	20/77	NA	NA	42	NA	NA
Donkol, 2013	18/8/NA	NA	NA	NA	31.1 ± 8.4	33.1 ± 7.5	NA
Zuhur, 2012	24/30/25	NA/24	NA	NA/25	29.1 ± 3.5	NA	30.8 ± 7.1
Kumar, 2009	34/31/NA	10/24	4/27	NA	37.1	29.1	NA
Assem, 2022	42/27/30	12/30	6/21	NA	37 ± 10	37 ± 14	34 ± 7
Chen, 2012	168/52/30	22/146	7/45	4/26	40.96 ± 12.41	42.52 ± 13.23	40.13 ± 11.25
Li, 2022	26/36/30	4/22	2/34	12/18	9.73 ± 3.56	11.46 ± 2.94	8.06 ± 2.74
Kim, 2015	40/20/60	12/28	7/13	25/35	43.23 ± 14.56	41.7 ± 13.97	47.33 ± 15.59
Hiraiwa, 2013	68/33/25	15/53	6/27	7/18	41.40 ± 4.56	36.77 ± 3.37	34.40 ± 3.31
Zhao, 2012(1)	103/32/30	29/74	12/20	11/19	45.8 ± 13.6	43.0 ± 12.3	40.1 ± 11.2
Zhao, 2012(2)	118/51/30	42/76	18/33	11/19	39.6 ± 13.7	45.0 ± 12.1	40.1 ± 11.2
Uchida, 2010	44/13/54	15/29	3/10	12/42	42.1 ± 14.4	43.8 ± 17.0	40.2 ± 13.0
Uchida, 2016	59/35/NA	12/47	12/23	NA	44	43	NA

GD, Graves Disease Group; DT, Destructive Thyrotoxicosis; NA, Not Applicable.

### 3.2 Pairwise pooling comparison between ITA and STA subgroups

**Figure 3:**
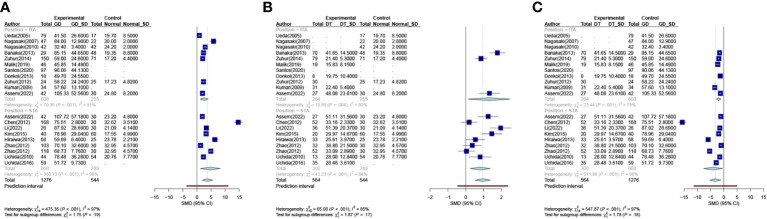
Meta-analysis of inter-group. (**(A)**. Group GD VS Group Normal **(B)**. Group DT VS Group Normal **(C)**. Group DT VS Group GD). GD Graves Disease Group, DT Destructive Thyrotoxicosis, SD Standard deviations, SMD, Standardized Mean Differences.

A subgroup analysis was conducted to assess the diagnostic utility of superior and inferior thyroid artery measurements. This analysis involved comparing the differences in STA-PSV (DSTA) and ITA-PSV (DITA) across various groups. In the comparison between the GD group and Normal group, the subgroup analysis of DSTA and DITA revealed no statistical significance (P = 0.19), as illustrated in **Figure 3A**. Similarly, comparisons between the DT group and the normal group, as well as between the GD and DT groups, showed no significant differences in DSTA and DITA, with P-values of 0.17 and 0.18, respectively (**Figures 3B, C**).

### 3.3 Post-pooling comparison of ITA and STA

**Figure 4:**
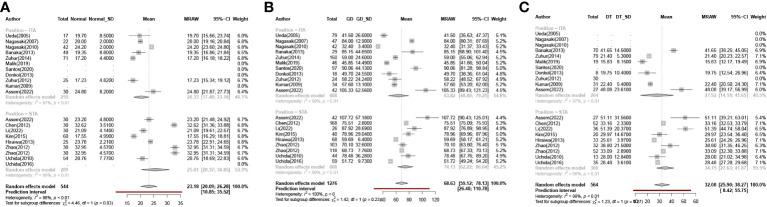
Meta-analysis of intra-group. (**(A)**. Group Normal **(B)** Group GD **(C)**. Group DT). GD Graves Disease Group, DT Destructive Thyrotoxicosis, SD Standard deviations, MRAW Mean of Raw data.

In the normal participant group, a statistically significant difference was observed between ITA-PSV and STA-PSV (P = 0.03), as shown in **Figure 4A**. The STA-PSV was higher in normal patients compared to ITA-PSV, with cut-off values of 20.33 cm/s (95% CI, 17.48-23.18) for ITA-PSV and 25.61 cm/s (95% CI, 20.37-30.85) for STA-PSV. To facilitate comparisons with other groups, we combined the data from the upper and lower thyroid arteries in normal participants, resulting in a combined cut-off value of 23.18 cm/s (95% CI, 20.09-26.28).

Contrastingly, in the GD and DT groups, the conditions were similar, as depicted in **Figures 4B, C**. No statistically significant difference was found between ITA-PSV and STA-PSV in these groups (PGD = 0.23; PDT = 0.27). In the GD group, the cut-off values were 63.82 cm/s (95% CI, 48.40-79.25) for ITA-PSV and 74.13 cm/s (95% CI, 62.22-86.04) for STA-PSV. In the DT group, these values were 27.92 cm/s (95% CI, 14.18-41.65) for ITA-PSV and 34.75 cm/s (95% CI, 27.63-41.87) for STA-PSV.

Consequently, the combined analysis of the upper and lower arteries revealed cut-off values for the peak systolic flow velocity of the thyroid arteries of 32.08 cm/s (95% CI, 25.90-38.27) and 68.63 cm/s (95% CI, 59.12-78.13) in individuals with DT and GD, respectively. This indicates that GD can be differentiated from DT by measuring the peak systolic velocity of the thyroid artery.

**Figure 5:**
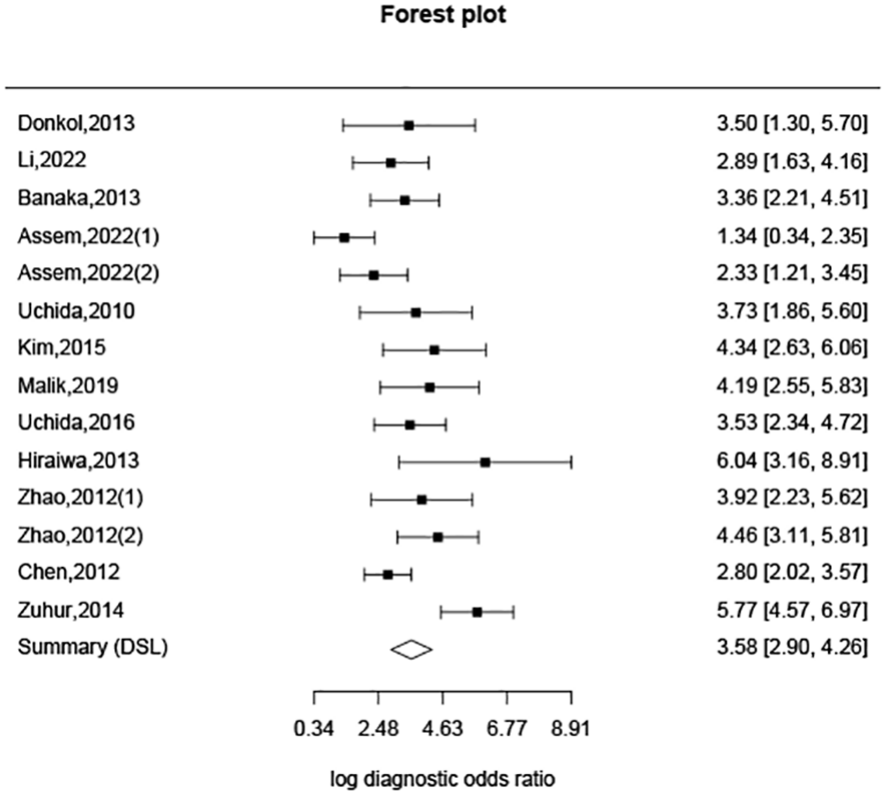
Diagnostic odds ratio estimation.

**Figure 6:**
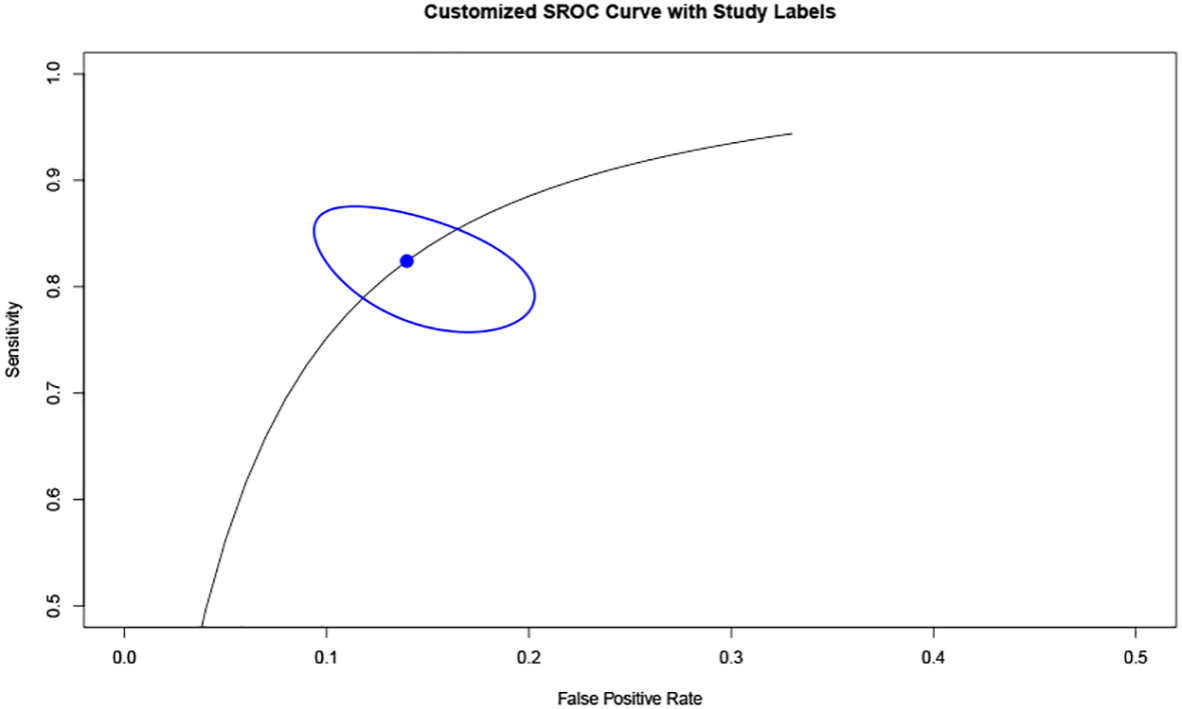
SROC curve. Summary Receiver Operating Characteristic (SROC) curve.

Additionally, based on sensitivity and specificity, a diagnostic odds ratio estimate (DSL) was calculated (**Table 2**, **Figure 5**), affirming that peak systolic flow velocity in the thyroid artery is a robust discriminator between GD and DT (DOR = 35.865). The summary receiver operating characteristic (SROC) curve (**Figure 6**) further corroborates the high overall diagnostic accuracy (AUC = 0.91), with a sensitivity estimate of 0.842 (95% CI, 0.772-0.866) and a false positive rate estimate of 0.140 (95% CI, 0.102-0.189).

**Table 2:** Diagnostic odds ratio estimation and confidence interval.

	DSL	2.5%	97.5%
DOR	35.865	18.218	70.603
lnDOR	3.58	2.902	4.257
tua²	1.112	0.000	2.222
tua	1.055	0.000	1.491

DOR Diagnosis Odds Ratio, DSL DerSimonian and Laird.

## 4 Discussion

In this comprehensive study, we encompassed a diverse cohort of 1276 patients diagnosed with GD, 564 individuals with DT, and 544 normative subjects, hailing from a wide array of nations including China, Japan, Korea, Egypt, Greece, Brazil, India, Turkey, and various Arabian countries. Our analysis focused on discerning the diagnostic thresholds of peak systolic flow velocities (PSV) in thyroid arteries, a pivotal factor in differentiating between these thyroid conditions. Our findings indicated that the cut-off for Inferior Thyroid Artery Peak Systolic Velocity (ITA-PSV) was 63.82 cm/s, and for Superior Thyroid Artery Peak Systolic Velocity (STA-PSV) was 74.13 cm/s in the GD cohort. Conversely, these values stood markedly lower in the DT group, at 27.92 cm/s for ITA-PSV and 34.75 cm/s for STA-PSV. Notably, the statistical analysis revealed no significant disparity between these groups, as evidenced by the P-value. This led us to amalgamate the data, resulting in threshold values of 68.63 cm/s for ITA-PSV and 32.08 cm/s for STA-PSV. In contrast, the normal subjects exhibited considerably lower cut-off values, with ITA-PSV at 20.33 cm/s and STA-PSV at 25.61 cm/s. Intriguingly, the within-group difference for this cohort was statistically significant, with a P-value of 0.03. These results underscore a pronounced disparity in peak systolic flow velocities in thyroid arteries among normal individuals, patients with GD, and those with DT. Specifically, the GD group manifested significantly higher thyroid artery PSV than both the DT group and the normative subjects. These observations align with the research conducted by Bogazzi et al. (36) and Hari Kumar et al. (10).

In light of these findings, we delved deeper into the cut-off value for peak systolic flow velocity in thyroid arteries among patients with GD and DT. By integrating the data from the superior and inferior thyroid arteries of the normal group, we established a combined cut-off value of 23.18 cm/s. This analysis elucidates a gradient in PSV values, with the GD group exhibiting the highest, followed by the DT group, and the normal subjects presenting the lowest values.

The utilization of peak flow velocity measurements in either the superior or inferior thyroid artery has been previously posited as an effective diagnostic tool for thyrotoxicosis (5, 35). This methodology has shown promise in distinguishing GD thyrotoxicosis from DT (4, 10, 15), as well as in monitoring the therapeutic response in GD patients post-radioactive iodine treatment (27). The value of STA-PSV has been equated to Radioactive Iodine Uptake (RAIU) in differentiating newly diagnosed thyroid toxicities (11, 31). ITA-PSV has been identified as a potential predictor for the early recurrence of GD following the cessation of Antithyroid Drug (ATD) therapy (14). Additionally, for differentiating GD and thyroiditis, ITA-PSV can serve as a supplementary method alongside TRAb and high-technique uptake of Tc-99m (16). However, these methodologies are not without their limitations. The inferior thyroid artery is more commonly measured in clinical settings (10) and exhibits significant anatomical variation. Conversely, the superior thyroid artery, while more superficially located, presents challenges in accurately measuring blood flow velocity due to changes in the flow direction, vascular lumen size, and spectrum (37, 38). Our research contributes to this field by enhancing the selectability and accuracy of data measurement by ultrasonographers for individual patients. This improvement helps circumvent the challenges posed by the anatomical location of the thyroid arteries, their variations, and other patient-specific differences. Our findings also provide new reference indices and diagnostic insights for clinical practice.

Moreover, our analysis reveals that the 95% confidence intervals (CI) for DT and GD do not overlap, indicating a clear distinction between the two groups based on cut-off values. While the difference in cut-off values between subjects with DT and normal subjects exists, it is not as pronounced as that between DT and GD. The overlap of 95% CI between DT and normal subjects may be attributable to variations between the upper and lower thyroid arteries in normal subjects, as well as individual differences encountered in clinical practice. The Diagnostic Odds Ratio (DOR) of 35.865, derived from analyzing the specificity and sensitivity differences between DT and GD, underscores the efficacy of this diagnostic approach. A DOR of 1 implies no discernible difference between individuals with and without GD. The higher the DOR, the more effective the diagnostic test. In our study, a DOR of 35.865 indicates a robust discriminatory capability. The Summary Receiver Operating Characteristic (SROC) curve, with an area under the curve (AUC) of 0.91, further corroborates the high overall diagnostic accuracy of this method. This substantiates the utility of peak systolic velocity measurements in thyroid arteries as a reliable means to differentiate between GD and DT.

The presence of significant heterogeneity in the pooling of STA-PSV and ITA-PSV across groups with DT, GD, and normal subjects was a notable concern in our study. This heterogeneity suggests considerable variability in STA-PSV and ITA-PSV between any two of these groups. Factors contributing to this variability could include differences in sample size, geographic and racial diversity of subjects (39), lifestyle factors, the technique employed by the sonographer, and the frequency of the ultrasound transducer used. This heterogeneity was also evident within the three groups themselves. Furthermore, our analysis, based on the chi-square (I²) value of subgroup differences, revealed that only the normal subjects exhibited significant differences between ITA-PSV and STA-PSV. In contrast, no statistically significant difference was observed between ITA-PSV and STA-PSV in either the GD or the DT groups. This finding suggests that, in cases of DT or GD, the ultrasonographic examination of either ITA-PSV or STA-PSV does not significantly impact diagnostic outcomes. This is likely due to both conditions causing an increase in blood flow velocity (40). In practical clinical settings, this allows for the selection of more easily measurable blood vessels, thereby enhancing the accuracy of measurements, expediting examinations, and improving overall efficiency.

However, our study is not without limitations. The predominance of Asian participants may limit the generalizability of our findings to other populations. Additionally, pregnant women with GD were not included in our subject pool. For this demographic, a combination of laboratory testing, clinical manifestations, and ultrasonography blood flow velocity detection should be employed for diagnosis. Further research is needed to evaluate the role of peak systolic flow velocity of thyroid arteries in diagnosing GD, potentially through multi-center collaborations.

In summary, our study establishes the utility of STA-PSV and ITA-PSV measurements in differentiating between GD and DT, with a diagnostic effectiveness of 91% when the threshold exceeds 68.63 cm/s for GD. We also determined that there is no significant difference in the measurement of upper and lower thyroid arteries in patients with either GD or DT. This finding enhances diagnostic precision, broadens the scope of measurement options for ultrasound technicians facing difficulties in measuring STA-PSV and ITA-PSV, and provides a novel reference index for the clinical differentiation of DT and GD.

## Author contributions

SL: Formal analysis, Methodology, Writing – original draft, Writing – review & editing. ZD: Software, Validation, Writing – original draft, Writing – review & editing. XL: Methodology, Resources, Writing – original draft, Writing – review & editing. MF: Data curation, Validation, Writing – review & editing. LS (5th author): Conceptualization, Investigation, Project administration, Writing – review & editing. MY: Project administration, Validation, Visualization, Writing – review & editing. RT: Methodology, Resources, Writing – review & editing. TG: Investigation, Supervision, Writing – review & editing. LS (9th author): Project administration, Supervision, Writing – original draft, Writing – review & editing.
