# Supplementary material for: A meta-analysis: elucidating diagnostic thresholds of peak systolic flow velocities in thyroid arteries for the discrimination of Graves’ disease and destructive thyrotoxicosis
Source: Front Endocrinol (Lausanne). 2024 Jun 7;15:1393126. doi: 10.3389/fendo.2024.1393126 (PMC11190173; doi:10.3389/fendo.2024.1393126)
Supplement: Supplementary file 1 [file DataSheet_1.pdf]

Supplementary Materials

Author/Year	Liner array transducer	Equipment	ITA-PSV（cm/s）					
			GD	DT	Normal	Sensitivity	specificity	Cut-off
Ueda,2005	7.5	Aloka,Japan	41.5±26.6	-	19.7±8.5	-	-	-
Nagasaki,2007	5	Aloka,Japan	84.0±12.9	-	20.0±2.0	-	-	-
Nagasaki,2010	5	Aloka，Japan	32.4±3.4	-	24.2±2.0	-	-	-
Banaka,2013	6-11	LOGIQBOOK XP,USA	85.15±44.65	41.65±14.5	19.35±8.8	0.828	0.869	61.65
Zuhur,2014	7-14	Toshiba,Japan	59.0±24.6	21.4±5.3	17.2±4.4	0.953	0.949	30
Malik,2019	7.5	Aloka,Japan	45.85±14.49	15.83±8.15	-	0.91	0.89	30
Santos,2020	5-12/8-14	Phillips,USA	90.06±44.13	-	-	-	-	-
Donkol,2013	3-9	Phillips,USA	49.7±24.55	19.75±10.4	-	0.889	0.875	40
Zuhur,2012	7-14	Toshiba,Japan	58.22±24.24	-	17.23±4.82	-	-	-
Kumar,2009	7.5	Philips,USA	57.6±13.1	22.4±5.4	-	-	-	-
Assem,2022	7-12	Phillips,USA	105.33±52.56	48.07±23.61	24.8±8.2	0.762	0.878	62.12

Supplementary table 1.Ultrasound results of ITA-PSV (inferior thyroid artery systolic peak flow rate) in subjects with GD Graves' disease

Author/Year	Liner array transducer	Equipment	STA-PSV（cm/s）					
			GD	DT	Normal	Sensitivity	specificity	Cut-off
Assem,2022	7-12	Philips,USA	107.72±57.18	51.11±31.56	23.2±4.8	0.667	0.667	76.57
Chen,2012	7.5	LOGIQ,USA	75.51±2.80	33.16±2.33	32.62±3.51	0.804	0.814	45.25
Li,2022	3-11	Mindray Resona7,China	87.92±28.69	51.39±20.37	21.09±4.14	0.769	0.861	64.64
Kim,2015	5-12	Philips, USA	78.96±29.04	29.97±14.67	17.55±4.99	0.95	0.85	41.3
Hiraiwa,2013	7.5-8	Tochigi,Japan	59.69±6.40	25.61±3.97	23.78±2.21	0.87	1	43
Zhao,2012（1）	7.5	Aloka A10	70.1±32.6	38.8±21.5	32.95±4.57	0.7087	0.9688	50
Zhao,2012（2）	7.5	Aloka A10	68.73±7.76	33.09±2.89	32.95±4.57	0.8163	0.9608	50
Uchida,2010	10	Hitachi,Japan	78.48±36.28	28±12.84	20.76±7.77	0.837	0.923	45
Uchida,2016	12	LOGIQ,USA	50.6（35.2-80.1）	28.0（22.6-37.8）	-	0.838	0.9	40

Supplementary table 2.Ultrasonic examination of STA-PSV (peak systolic flow rate of superior thyroid artery) in subjects with GD Graves disease, (1) retrospective study, (2) prospective study

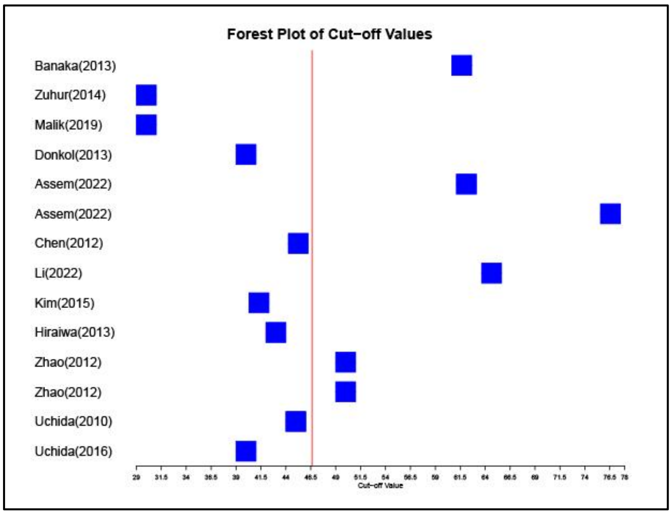

Supplementary figure 1.Forest map of ITA/STA-PSV Cut-off values of subjects tested by ultrasound
